# Safety, Pharmacokinetics, and Activity of High-Dose Ivermectin and Chloroquine against the Liver Stage of Plasmodium cynomolgi Infection in Rhesus Macaques

**DOI:** 10.1128/AAC.00741-20

**Published:** 2020-08-20

**Authors:** Pattaraporn Vanachayangkul, Rawiwan Im-erbsin, Anchalee Tungtaeng, Chanikarn Kodchakorn, Alison Roth, John Adams, Chaiyaporn Chaisatit, Piyaporn Saingam, Richard J. Sciotti, Gregory A. Reichard, Christina K. Nolan, Brandon S. Pybus, Chad C. Black, Luis A. Lugo-Roman, Matthew D. Wegner, Philip L. Smith, Mariusz Wojnarski, Brian A. Vesely, Kevin C. Kobylinski

**Affiliations:** a Department of Bacterial and Parasitic Diseases, Armed Forces Research Institute of Medical Sciences, Bangkok, Thailand; b Department of Veterinary Medicine, Armed Forces Research Institute of Medical Sciences, Bangkok, Thailand; c Department of Drug Discovery, Experimental Therapeutics Branch, Walter Reed Army Institute of Research, Silver Spring, Maryland, USA; d Center for Global Health and Infectious Diseases Research, College of Public Health, University of South Florida, Tampa, Florida, USA; e Department of Entomology, Armed Forces Research Institute of Medical Sciences, Bangkok, Thailand

**Keywords:** COVID-19, *Plasmodium*, SARS-CoV-2, chloroquine, ivermectin, macaque, pharmacokinetics, safety

## Abstract

Previously, ivermectin (1 to 10 mg/kg of body weight) was shown to inhibit the liver-stage development of Plasmodium berghei in orally dosed mice. Here, ivermectin showed inhibition of the *in vitro* development of Plasmodium cynomolgi schizonts (50% inhibitory concentration [IC_50_], 10.42 μM) and hypnozoites (IC_50_, 29.24 μM) in primary macaque hepatocytes when administered as a high dose prophylactically but not when administered in radical cure mode. The safety, pharmacokinetics, and efficacy of oral ivermectin (0.3, 0.6, and 1.2 mg/kg) with and without chloroquine (10 mg/kg) administered for 7 consecutive days were evaluated for prophylaxis or radical cure of P. cynomolgi liver stages in rhesus macaques. No inhibition or delay to blood-stage P. cynomolgi parasitemia was observed at any ivermectin dose (0.3, 0.6, and 1.2 mg/kg). Ivermectin (0.6 and 1.2 mg/kg) and chloroquine (10 mg/kg) in combination were well-tolerated with no adverse events and no significant pharmacokinetic drug-drug interactions observed. Repeated daily ivermectin administration for 7 days did not inhibit ivermectin bioavailability. It was recently demonstrated that both ivermectin and chloroquine inhibit replication of the novel severe acute respiratory syndrome coronavirus 2 (SARS-CoV-2) *in vitro*. Further ivermectin and chloroquine trials in humans are warranted to evaluate their role in Plasmodium vivax control and as adjunctive therapies against COVID-19 infections.

## Abstract (precis)

Previously, ivermectin (1 to 10 mg/kg of body weight) was shown to inhibit the liver-stage development of Plasmodium berghei in orally dosed mice. Here, ivermectin showed inhibition of the *in vitro* development of Plasmodium cynomolgi schizonts (50% inhibitory concentration [IC_50_], 10.42 μM) and hypnozoites (IC_50_, 29.24 μM) in primary macaque hepatocytes when administered as a high dose prophylactically but not when administered in radical cure mode.

## TEXT

Novel chemoprophylactic therapeutics and vector control interventions could support and accelerate malaria elimination efforts. Ivermectin mass drug administration (MDA) has been proposed as a malaria control tool since it makes the blood of treated persons lethal to *Anopheles* mosquitoes, the vectors of malaria (1–5), and repeated ivermectin MDAs in Burkina Faso were able to reduce malaria transmission to humans (6). Ivermectin is a safe and well-tolerated endectocidal drug used widely in veterinary and human medicine to combat both internal and external parasites.

Ivermectin has been shown to inhibit the liver-stage development of Plasmodium berghei in both an *in vitro* Huh7 human hepatoma cell line model (7) and an *in vivo* C57BL/6 mouse model (8). The *in vitro* 50% inhibitory concentration (IC_50_) for ivermectin P. berghei inhibition, 1.8 μg/ml (2.1 μM), was higher than blood levels that can be achieved in treated humans. However, mice that were orally dosed with ivermectin at 1 to 10 mg/kg of body weight at 24 and 12 hours before and 12 hours after sporozoite challenge demonstrated liver-stage inhibition equal to treatment with primaquine (10 mg/kg) under the same dosing schedule (8). Human equivalent dosing (HED) that was evaluated in mice would correlate to ivermectin doses in the range of 0.08 to 0.81 mg/kg (9). Thus, ivermectin is promising for human malaria chemoprophylaxis, as ivermectin doses as high as 2 mg/kg have been safely administered to humans (10). If ivermectin can prevent *Plasmodium* liver-stage infection, then ivermectin chemoprophylaxis could be considered for use in high-risk groups, such as forest-goers in the Greater Mekong Subregion or naive soldiers deployed to areas of malaria endemicity. Furthermore, if ivermectin MDA is deployed for community-wide malaria vector control and if ivermectin is chemoprophylactic, then there would be direct benefits to MDA participants in preventing malaria infections.

Plasmodium cynomolgi infections in rhesus macaques (Macaca mulatta) are routinely used as a surrogate human liver-stage model for Plasmodium vivax drug development. This model can evaluate both the causal prophylaxis, (i.e., protection from developing liver schizonts) and the hypnozoiticidal (i.e., radical cure of liver hypnozoites) efficacy of compounds (11). Ivermectin has been used in rhesus macaque colonies to treat mites (12), lice (13), and intestinal helminths, such as *Ascaris* sp., *Trichuris* sp., and Strongyloides fuelleborni (14–16). Preclinical studies demonstrated that oral ivermectin was safe in macaques at doses up to 1.2 mg/kg for 14 days and that macaques are an ideal animal model for ivermectin human treatment (17, 18). However, no study to date has evaluated the pharmacokinetics of repeated ivermectin treatment in rhesus macaques or in combination with chloroquine.

Here, we evaluate the *in vitro* and *in vivo* liver-stage effect of ivermectin against P. cynomolgi in rhesus macaque liver hepatocytes and infected macaques. The safety and pharmacokinetics of repeated oral ivermectin dosing with and without chloroquine in macaques are also presented.

## RESULTS

### *In vitro* results.

**FIG 1:**
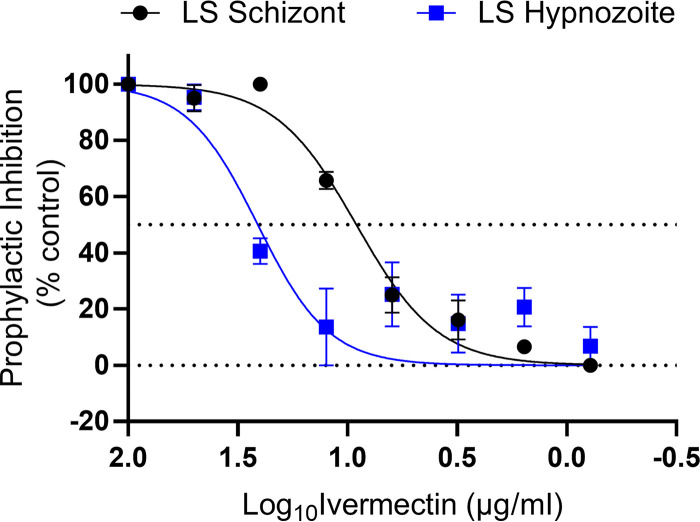
*In vitro* P. cynomolgi liver-stage ivermectin inhibition prophylactic results. Prophylactic (days 1 to 3) exposure of P. cynomolgi to ivermectin demonstrated marginal inhibition of liver schizonts (IC_50_, 9.12 μg/ml) and hypnozoites (IC_50_, 25.59 μg/ml). LS, liver stage. Graph bars represent means with standard deviation of biological replicates (*n* = 3) with experimental replicates (*n* = 2).

Ivermectin efficacy against liver-stage parasites was initially evaluated using an *in vitro* P. cynomolgi liver model which utilizes primary rhesus macaque hepatocytes in order to closely resemble the *in vivo* antirelapse mode. The drugging regimen was defined by treatment mode, which was either a prophylactic mode (i.e., drug administered with sporozoites and 3 days thereafter) or a radical cure mode (i.e., drug administered from days 4 to 7 post-sporozoite infection) similar to that in previously described methods (19). In prophylactic mode, ivermectin showed marginal *in vitro* causal protection against the development of P. cynomolgi-infected rhesus macaque hepatocyte liver schizonts (IC_50_, 9.12 μg/ml; 10.42 μM) and hypnozoites (IC_50_, 25.59 μg/ml; 29.24 μM) (Fig. 1). However, in radical cure mode, ivermectin had no activity on developing P. cynomolgi liver schizonts or established hypnozoites, even when dosed at a high initial concentration of 100 μg/ml (114.26 μM).

### *In vivo* results for ivermectin and chloroquine safety and tolerability.

There was only one adverse event in a single macaque (R1435) that vomited 3 hours after the first oral dose of ivermectin (1.2 mg/kg) when administered as monotherapy 1 day prior to P. cynomolgi sporozoite injection. No adverse events occurred when ivermectin (0.6 or 1.2 mg/kg) was coadministered with chloroquine. No abnormal hematology outcomes were observed for ivermectin alone or ivermectin plus chloroquine coadministration.

### *In vivo* results for parasitemia.

**FIG 2:**
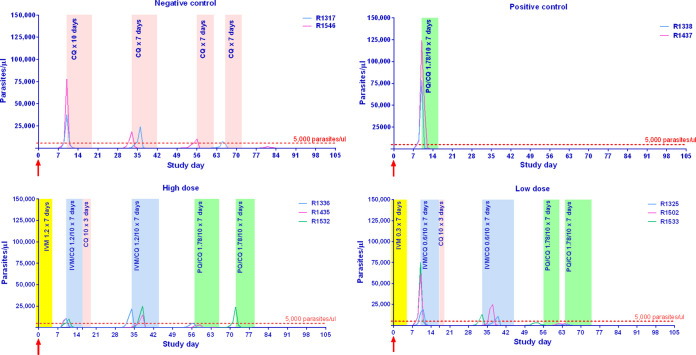
Blood-stage P. cynomolgi parasitemia results and drug regimens for each treatment group. Displays the number of P. cynomolgi blood-stage parasites per μl of blood observed via microscopy. Shaded areas represent the duration of drug administration when daily drug dosing was administered, with yellow for ivermectin, peach for chloroquine, blue for ivermectin plus chloroquine, and green for primaquine plus chloroquine. Drug concentrations are stated as numbers, and all quantities displayed are in mg/kg. Numbers (i.e., Rxxx) in the legend denote the individual macaque identification number. The red arrows indicate when sporozoites were administered. The dashed red line denotes the 5,000 parasites per μl cutoff to trigger drug administration. IVM, ivermectin; CQ, chloroquine; PQ, primaquine.

Primary blood-stage parasitemia greater than 5,000/μl was detected 10 days postinoculation for negative- and positive-control groups and for 2 of 3 macaques in both ivermectin high-dose (1.2 mg/kg) and low-dose (0.3 mg/kg) groups, with the remaining macaques from each group reaching greater than 5,000/μl at 11 days postinoculation, which was 5 and 6 days after the last ivermectin administration, respectively. Primary infection blood-stage parasitemia was cleared from the negative-control group with 10 days of chloroquine (10 mg/kg), and both blood- and liver-stage parasites were cleared from the positive-control group with 7 days of chloroquine (10 mg/kg) and primaquine (1.78 mg/kg). Blood-stage parasitemia was cleared from the three macaques in the low-dose ivermectin group with 7 days ivermectin (0.6 mg/kg) and 10 days chloroquine (10 mg/kg). Two of three macaques were cleared of primary infection blood-stage parasitemia in the high-dose group with ivermectin (1.2 mg/kg) for 7 days and chloroquine (10 mg/kg) for 10 days, while one macaque was cleared with ivermectin (1.2 mg/kg) and chloroquine (10 mg/kg) for 7 days. However, the first relapse occurred within 3 weeks, at approximately the same time for negative-control and both ivermectin groups, with no significant differences for time to blood-stage parasitemia or treatment (log-rank [Mantel Cox] test, *P* > 0.05). The first relapse infection blood-stage parasitemia was cleared from the negative control with chloroquine (10 mg/kg) alone for 7 days. First relapse infection blood-stage parasitemia was cleared from both high-dose (1.2 mg/kg) and low-dose (1.2 mg/kg) ivermectin groups when given in combination with chloroquine (10 mg/kg) for 7 days. Approximately 3 weeks later, a second relapse occurred in all negative-control and ivermectin high- and low-dose-treated macaques with no significant differences for time to blood-stage parasitemia or treatment (log-rank [Mantel Cox] test, *P* > 0.05). At the point of second relapse, all ivermectin group macaques were treated with primaquine (1.78 mg/kg) and chloroquine (10 mg/kg) for 7 days. The positive-control group was treated with primaquine (1.78 mg/kg) and chloroquine (10 mg/kg) for 7 days at the point of primary infection and had no relapses for the remainder of the study (Fig. 2). The negative-control group was treated with primaquine (1.78 mg/kg) and chloroquine (10 mg/kg) for 7 days at the point of third relapse (data not shown).

The real-time PCR (RT-PCR) method detected primary blood-stage parasitemia 1 day earlier than microscopy at the point of first infection for the negative- and positive-control group macaques and in two out of three ivermectin low-dose (0.3 mg/kg) macaques. The remaining four ivermectin high- and low-dose macaques had blood-stage parasitemia detected by RT-PCR on the same day as microscopy.

### *In vivo* results for pharmacokinetics.

**FIG 3:**
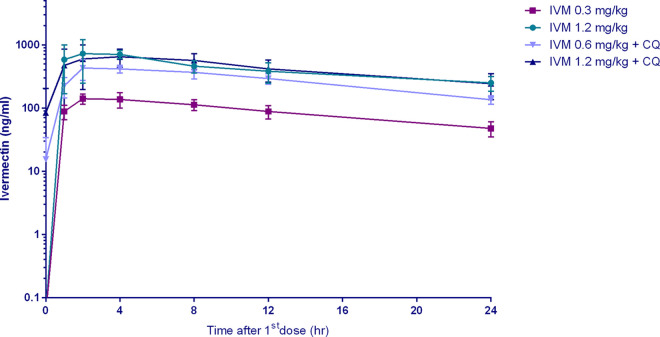
Ivermectin concentrations achieved in macaques. Represents the log concentration of ivermectin achieved in orally dosed macaques within 24 hours after the first dose. IVM, ivermectin; CQ, chloroquine (10 mg/kg).

Plasma ivermectin with and without coadministration of 10 mg/kg chloroquine reached maximum concentration of drug in serum (*C*_max_) at approximately 2 to 4 hours postdose, and the elimination half-life ranged from 11 to 28 hours with an accumulation index of 0.6 to 3.7. The plasma concentration time profile for the first 24 hours and pharmacokinetic parameters of ivermectin are shown in Fig. 3 and Tables 1 and 2.

**TABLE 1:** Pharmacokinetic parameters of ivermectin alone after 1st and 7th dose described by noncompartmental analysis^*a*^

PK parameter	Ivermectin 0.3 mg/kg				Ivermectin 1.2 mg/kg			
	1^st^ dose		7^th^ dose		1^st^ dose		7^th^ dose	
	Avg	SD	Avg	SD	Avg	SD	Avg	SD
AUC_%Extrap_ (%)	29.2	4.1	17.6	11.5	37.4	17.8	19.1	19.1
AUC_24_ (h × ng/ml)	2,152	460	6,481	853	10,188	2,781	28,495	7,190
AUC_∞_ (h × ng/ml)	n/a	n/a	8,017	1,950	n/a	n/a	37,609	17,783
CL/F (liter/h/kg)	0.10	0.02	0.04	0.01	0.08	0.02	0.04	0.01
*V_z_*/F (liter/kg)	1.90	0.41	1.03	0.25	1.73	0.61	1.15	0.16
*C*_max_ (ng/ml)	145.0	27.7	341.0	117.4	865.3	246.0	984.3	92.1
*C*_max_/dose (kg × ng/ml/mg)	483.3	92.4	1,136.7	391.3	721.1	205.0	820.3	76.8
*t*_1/2_ (h)	13.1	1.9	19.2	6.9	16.5	6.0	24.1	8.3
*T*_max_ (h)	3.3	1.2	6.7	4.6	2.7	1.2	5.3	2.3

a The pharmacokinetic parameters of ivermectin when administered alone after the first and seventh (last) doses. AUC_%Extrap_, the percentage of area under the concentration-time curve after the last dose to infinity due to extrapolation from last collection time point; AUC_24_, area under the concentration-time curve in 24 hours; AUC_∞_, area under the concentration-time curve after the last dose to infinity (the total exposure); CL/F, the apparent clearance; *V_z_*/F is the apparent volume of distribution during terminal phase; *C*_max_, the maximum concentration of drug in serum; *C*_max_/dose, the maximum concentration divided by the dose administered; *t*_1/2_ is the elimination half-life; *T*_max_ is the time to reach *C*_max_; n/a, not applicable.

**TABLE 2:** Pharmacokinetic parameters of ivermectin when coadministered with chloroquine after 1st and 7th dose described by noncompartmental analysis^*a*^

PK parameter	Ivermectin 0.6 mg/kg + CQ 10 mg/kg				Ivermectin 1.2 mg/kg + CQ 10 mg/kg			
	First dose		Last dose		First dose		Last dose	
	Avg	SD	Avg	SD	Avg	SD	Avg	SD
AUC_%Extrap_ (%)	24.8	6.4	1.3	1.1	30.4	13.5	4.5	5.7
AUC_24_ (h × ng/ml)	6,742	415	18,333	8,989	10,406	2,793	38,079	41,331
AUC_∞_ (h × ng/ml)	n/a	n/a	18,618	9,314	n/a	n/a	39,184	42,094
CL/F (liter/h/kg)	0.07	0.01	0.04	0.01	0.09	0.03	0.06	0.05
*V_z_*/F (liter/kg)	1.10	0.19	1.20	0.29	1.55	0.47	1.60	0.75
*C*_max_ (ng/ml)	493.3	62.1	419.7	21.5	742.0	256.1	942.3	266.1
*C*_max_/dose (kg × ng/ml/mg)	822.2	103.5	699.4	35.8	618.3	213.4	785.3	221.7
*t*_1/2_ (h)	11.5	2.5	25.3	12.1	13.3	4.5	28.3	23.6
*T*_max_ (h)	4.0	3.5	4.0	0.0	4.0	3.5	4.0	0.0

a The pharmacokinetic parameters of ivermectin when administered with chloroquine (10 mg/kg) after the first and seventh (last) doses. AUC_%Extrap_, the percentage of area under the concentration-time curve after the last dose to infinity due to extrapolation from last collection time point; AUC_24_, area under the concentration-time curve in 24 hours; AUC_∞_, area under the concentration-time curve after the last dose to infinity (the total exposure); CL/F, the apparent clearance; *V_z_*/F is the apparent volume of distribution during terminal phase; *C*_max_, the maximum concentration of drug in serum; *C*_max_/dose, the maximum concentration divided by the dose administered; *t*_1/2_ is the elimination half-life; *T*_max_ is the time to reach *C*_max_; CQ, chloroquine; n/a, not applicable.

## DISCUSSION

Ivermectin alone was safe and well-tolerated in macaques with repeated doses at 0.3 and 1.2 mg/kg for 7 days, with no signs of neurological, gastroenterological, or hematological complications. One monkey vomited the first dose of ivermectin (1.2 mg/kg) when administered as monotherapy but had no emesis upon further dosing. Emesis was observed previously in ivermectin-treated macaques receiving a 2-mg/kg single dose, and the occurrence of emesis increased with higher doses (4, 6, 8, 12, and 24 mg/kg) (17, 18). The combination of ivermectin (0.6 and 1.2 mg/kg) and chloroquine (10 mg/kg) for 7 days was safe and well-tolerated in macaques. This finding suggests that this combination could be used in humans during P. vivax MDAs in regions where chloroquine is still an effective P. vivax blood-stage therapeutic.

Prophylactic mode *in vitro* results with an ivermectin parent compound indicated ivermectin activity against P. cynomolgi liver schizonts and hypnozoites (Fig. 1) but at higher concentrations than could be safely achieved in humans (10). However, there is a growing body of evidence that the activity of ivermectin is not restricted to the parent compound alone and that ivermectin metabolites may be active as well. Indeed, when comparing the effect of ivermectin metabolized by a human to that of parent compound mixed in human blood, the mosquito-lethal effect against Anopheles dirus and Anopheles minimus was 20- to 35-fold more potent (5) and the sporontocidal effect against P. vivax development in Anopheles aquasalis was 5-fold more potent (20). Even though P. berghei *in vitro* liver-stage IC_50_s were in the μg/ml range, liver schizont inhibition was achieved *in vivo* with ivermectin at doses plausible for use in humans (8). The points above warranted evaluation of ivermectin against P. cynomolgi in rhesus macaques even though *in vitro* IC_50_s were in the μg/ml range and ivermectin reaches only ng/ml concentrations in orally treated hosts.

There was no delay to patency of first blood-stage P. cynomolgi infection in either low- or high-dose ivermectin groups (Fig. 2). Ivermectin displayed μM levels of liver schizont efficacy *in vitro*; however, a lack of delay to blood-stage patency suggests a minimal impact of ivermectin on liver schizont development. Admittedly, the injection of one million P. cynomolgi sporozoites into the macaque sets a very high bar for any drug, as it only requires one sporozoite to develop into a liver schizont to continue the blood-stage malaria infection. This is in contrast to a single mosquito that is predicted to deliver <100 sporozoites during blood feeding (21). The *in vitro* ivermectin experiments indicated prophylactic inhibition of P. cynomolgi hypnozoite development at μM concentrations; however, the macaque ivermectin challenge clearly demonstrated development of hypnozoites, as indicated by the first and second blood-stage relapses occurring at approximately the same time as negative vehicle controls (Fig. 2). Neither *in vitro* nor *in vivo* P. cynomolgi models indicate a radical cure efficacy potential for ivermectin. A recent human challenge trial (*n* = 8) with intravenous injection of cryopreserved Plasmodium falciparum sporozoites (*n* = 3,200) and a single oral dose ivermectin (400 μg/kg) failed to show liver-stage inhibition in terms of time to blood-stage patency (22).

**FIG 4:**
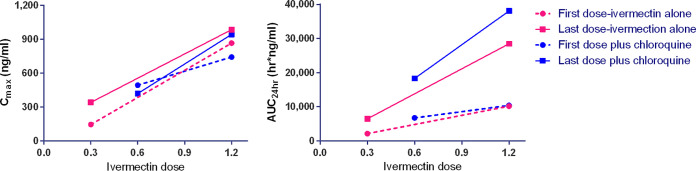
Pharmacokinetic simulation of ivermectin concentration-time profile when given at 0.3, 0.6, and 1.2 mg/kg for 7 days in rhesus macaques. Illustrates the simulation of plasma ivermectin concentration-time profile. One-compartment analysis best described the observed data by using the estimates calculated by noncompartmental analysis following the first and seventh doses as initial estimates. In the simulation, *C*_max_ had mean estimates of 150, 300, and 600 ng/ml at approximately 4 h after the first dose and reached a steady state around the fifth dose with *C*_max_ at 243, 486, and 973 ng/ml at the ivermectin doses 0.3, 0.6, and 1.2 mg/kg, respectively. IVM, ivermectin.

**FIG 5:**
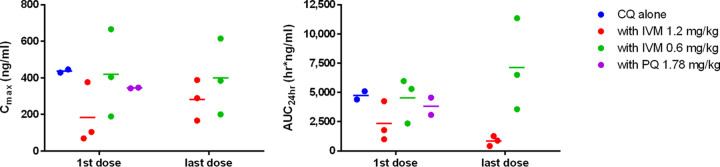
Mean plasma concentration-time profiles of ivermectin 24 hours after the first and seventh dose when administered ivermectin at 0.3, 0.6, and 1.2 mg/kg with and without chloroquine (10 mg/kg). Illustrates the mean ivermectin plasma concentration (ng/ml) by time (h) profile 24 hours after the first and seventh dose with or without CQ (10 mg/kg). There was a slight reduction in peak concentrations achieved and a delay in time to achieve peak concentrations when comparing the first and seventh doses. IVM, ivermectin; CQ, chloroquine.

To the best of our knowledge, this is highest repeated dose ivermectin pharmacokinetic investigation in any mammal species (Fig. 4). There were no significant changes in the clearance per fraction of drug absorbed (CL/F) or half-life (*t*_1/2_) values (Table 1 and Fig. 5). It should be noted that this study had a small sample size, with only three macaques per ivermectin-treated group, and thus, ivermectin autoinhibition warrants further evaluation in future trials. In humans, three repeated doses of ivermectin (30 or 60 mg) every third day did not inhibit *C*_max_ when comparing the first and third dose, suggesting a lack of autoinhibition (10). In FVB mice administered oral ivermectin (0.2 mg/kg) twice a week for 5 weeks, there was a 1.7-fold reduction in the 24-hour postdose plasma ivermectin concentrations, while the major metabolite concentration increased by 1.7-fold (23), suggesting an induction of metabolism.

**FIG 6:**
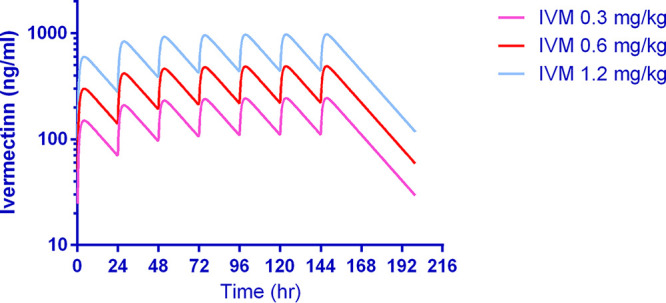
Relative ivermectin parameter values for *C*_max_ (left) and AUC_24_ (right). Illustrates the linear pharmacokinetics of ivermectin as *C*_max_ and AUC increased in a dose-dependent manner. Higher dose of ivermectin resulted in increased drug exposure with repeated dosing. Chloroquine did not interfere with ivermectin pharmacokinetics.

**FIG 7:**
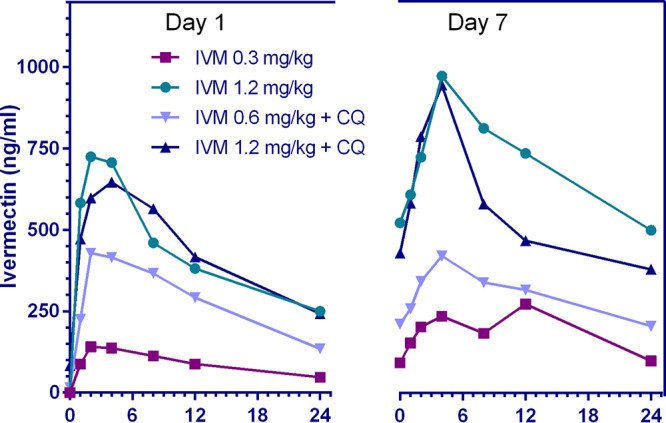
Relative chloroquine parameter values for *C*_max_ (left) and AUC_24_ (right). Illustrates that ivermectin did not have any effect on chloroquine *C*_max_ or AUC_24_ (paired sample *t* test, *P* > 0.05). IVM, ivermectin; CQ, chloroquine; PQ, primaquine.

In macaques, coadministration of ivermectin (0.6 or 1.2 mg/kg) and chloroquine (10 mg/kg) for 7 days was safe and well-tolerated. Coadministration of chloroquine and ivermectin did not have an effect on the *C*_max_ or area under the concentration-time curve (AUC) of ivermectin (Table 1 and 2 and Fig. 6) or chloroquine (Fig. 7). The 1.2- and 0.6-mg/kg doses in macaques have approximate human equivalent doses (HEDs) of 0.55 mg/kg (total, 3.85 mg/kg) and 0.27 mg/kg (total, 1.89 mg/kg), respectively. This suggests that repeated daily dosing of ivermectin at 0.6 or 0.3 mg/kg could be used in combination with chloroquine in humans. While billions of ivermectin and chloroquine treatments have been administered to humans, there is very limited safety evidence for their coadministration. Only one study, on P. vivax, has coadministered ivermectin (0.2 mg/kg single dose) and chloroquine (0.6 mg/kg on the first day, 0.45 mg/kg on the second and third day), and they did so in 10 persons with no adverse events passively reported (20).

Ivermectin (24), chloroquine (25), and hydroxychloroquine (26, 27) have been shown *in vitro* to inhibit replication of the novel severe acute respiratory syndrome coronavirus 2 (SARS-CoV-2). All three drugs distribute into lung tissues at higher concentrations than plasma for chloroquine and hydroxychloroquine in rats (28), for hydroxychloroquine in mice (29), and for ivermectin in goats (30) and cattle (31). The preclinical safety evidence in macaques presented here and *in vitro* efficacy warrant further investigation of ivermectin and chloroquine or hydroxychloroquine in SARS-CoV-2-infected persons.

This work verifies that the rhesus macaque model provides a robust system for evaluating ivermectin pharmacokinetics. Newer formulations of ivermectin in development for humans, such as implants and expandable pill formulations (32, 33), could be evaluated in rhesus macaques. Novel methods of Plasmodium knowlesi control, such as treatment of wild primates with ivermectin baits to target wild *Anopheles* populations, could potentially be evaluated in this ivermectin macaque model system.

Although ivermectin was able to inhibit the liver-stage development of P. cynomolgi *in vitro*, no demonstrable effect was observed with *in vivo* macaque challenge. Repeated doses of ivermectin (0.3, 0.6, and 1.2 mg/kg) for 7 days in macaques was safe, with a corresponding rise in drug exposures (AUC), but no signs of autoinhibition. Coadministration of ivermectin (0.6 or 1.2 mg/kg) and chloroquine was safe and well-tolerated, with no drug-drug interactions altering ivermectin or chloroquine pharmacokinetics. Further ivermectin and chloroquine trials in humans are warranted for P. vivax control and SARS-CoV-2 chemoprophylaxis and treatment.

## MATERIALS AND METHODS

### *In vitro* assay.

The complete methodology we used is pending publication (A. Roth, personal communication). In brief, cryopreserved primary nonhuman primate hepatocytes (lot NGB) and hepatocyte culture medium (HCM) (InVitroGro CP medium) were obtained from BioIVT, Inc., (Baltimore, MD, USA) and thawed following manufacturer recommendations. The hepatocytes were plated into collagen-coated 384-well plates and were used for experiments within 2 to 4 days after plating (19). Infectious sporozoites were obtained from *An. dirus* mosquitoes infected with P. cynomolgi B strain and were used to infect the plated primary nonhuman primate hepatocytes. Ivermectin compound (lot number MKBZ1802V; Sigma-Aldrich, St. Louis, MO, USA) was dissolved in 100% dimethyl sulfoxide (DMSO) and used at a final concentration of 100 μg/ml in an 8-point, 2-fold serial dilution. Ivermectin was administered in two treatment modes, prophylactic and radical cure. In prophylactic mode, the drug was present for 4 days, starting at the point of sporozoite addition. Alternatively, in radical cure mode, the drug was present for 4 days, starting on day 4 post-sporozoite inoculation (19, 34–36).

Imaging and data analysis of the drug plates were completed using the Operetta CLS imaging system and Harmony software 4.1 (Perkin Elmer, Waltham, MA, USA). Images were acquired using tetramethyl rhodamine isocyanate (TRITC), 4′,6-diamidino-2-phenylindole (DAPI), and bright field channels at ×10 magnification. Using similar methodology described previously, parasites were counted with the TRITC channel and were identified by area, mean intensity, maximum intensity, and cell roundness (19, 34–36). Ivermectin IC_50_ curves and percent inhibition were generated using parasite population counts where controls were calculated as the average of replicates. The reported IC_50_s were obtained from two experimental replicates with three biological replicates for prophylactic mode and two biological replicates for radical cure mode, using an 8-point concentration format with 2-fold dilutions for final ivermectin concentrations of 0.781 to 100 μg/ml. The percent inhibition was performed using dose-response modeling in Prism version 8.0 (GraphPad, La Jolla, CA, USA), where measured parasite quantity (hypnozoite or schizont parasites) was normalized to the negative control (infected wells) using the average of experimental and biological replicates.

### *In vivo* macaque trial.

Anopheles dirus mosquitoes were used to produce P. cynomolgi (B strain) sporozoites, from a donor macaque infected with blood-stage P. cynomolgi parasites. For the liver-stage challenge, each macaque was intravenously injected with 1 × 10^6^ P. cynomolgi sporozoites in a 1-ml inoculum of phosphate-buffered saline (PBS) and 0.5% bovine serum albumin. USAMD-AFRIMS colony-born rhesus macaques of Indian origin were used in this study. Ten healthy macaques, five males and five females, 3 to 5 years old, and ranging in weight from 4.5 to 6.4 kg, were selected for this study. All macaques were negative for simian retroviruses and simian herpes B virus. Two macaques served as negative controls and were treated initially with 7 days of vehicle controls, with 7 days of chloroquine (10 mg/kg) when parasitemia reached >5,000 parasites per μl at primary infection and first relapse, and with 7 days chloroquine (10 mg/kg) plus primaquine (1.78 mg/kg) at second relapse. Two macaques served as positive causal prophylaxis controls and were treated initially with 7 days of vehicle controls and treated with 7 days of chloroquine (10 mg/kg) plus primaquine (1.78 mg/kg) at the point of primary infection when parasites reached >5,000 parasites per μl. All study drugs were administered to restrained conscious macaques via nasogastric intubation at 1 ml/kg of body weight.

Sparmectin-E (Sparhawk Laboratories, Inc., Lenexa, KS, USA) is a water-soluble formulation of ivermectin developed for oral use in horses. Ivermectin was diluted in sterile water and administered via nasogastric route. Six macaques total received ivermectin, with three receiving low-dose (0.3 mg/kg) and three high-dose (1.2 mg/kg), for 7 consecutive days starting 1 day before sporozoite challenge. If a primary blood-stage infection occurred and blood-stage parasitemia reached >5,000 parasites per μl, then the macaques received 7 days of chloroquine (10 mg/kg) plus ivermectin (1.2 mg/kg) for the high-dose group and 7 days of chloroquine (10 mg/kg) plus ivermectin (0.6 mg/kg) for the low-dose group. If a relapse occurred and blood-stage parasitemia reached >5,000 parasites per μl, then macaques received 7 days of chloroquine (10 mg/kg) plus ivermectin (1.2 mg/kg) for both the low- and high-dose groups. If a second relapse occurred, then the macaques were treated with 7 days of chloroquine (10 mg/kg) and primaquine (1.78 mg/kg), terminating the experiment. Both the negative and positive-control group macaques were treated with 7 days of chloroquine (10 mg/kg) and primaquine (1.78 mg/kg) at the third relapse and first infection, respectively.

Macaques were observed several times in the first few hours after dosing and at least three times a day for the remainder of the study for any clinical signs of neurological (e.g., ataxia, lethargy, and imbalance) or gastroenterological (e.g., diarrhea, vomiting, and weight loss) complications. Venous blood was collected at select time points, and after macaques became blood smear positive for hematocrit, white and red blood cell counts were determined.

### Parasitemia monitoring.

**(i) Microscopy.** Thick and thin blood smear samples were made and examined daily to quantify malaria parasitemia. Thin smears were fixed in methanol and stained with Giemsa stain. Thick smears were stained with Giemsa stain without fixation. Blood smears were examined for the presence or absence of blood-stage parasites under a ×100 oil-immersion objective. If no parasites were found in 50 microscopic oil-immersion thick fields or approximately 1,000 white blood cells (WBCs), the smear was considered negative. The parasitemia level was reported as the number of parasites per 1 μl or mm^3^ of whole blood. Parasites were counted per number of WBCs or red blood cells (RBCs) (i.e., per 1,000 WBCs or 1,000 to 10,000 RBCs). Parasitemia levels were calculated by the appropriate total blood cell count (white or red) per mm^3^.

**(ii) Real-time PCR.** Blood samples (0.2 ml) were collected on days 5, 6, and 7 after sporozoite injection. The same sampling schedule occurred in control macaques, with the addition of sampling days 8, 9, and 10 (1 ml) to obtain infected blood for controls used for method development. Blood was collected, stored in EDTA tubes, and kept frozen at –80°C. Parasite DNA was extracted from 200 μl from EDTA whole blood using the EZ1 DNA blood kit with automated EZ1 advanced XL purification system (Qiagen, Hilden, Germany). Real-time PCR for P. cynomolgi detection was performed by using the Rotor Gene Q 5plex high-resolution melting (HRM) platform (Qiagen). A primer and probe were designed to target P. cynomolgi small subunit rRNA of blood-stage parasites (GenBank accession number L08242.1). Primers and probe names and sequences are as follows: P. cynomolgi Fwd, 5′-ATTGCGGTCGCAAATAATGAAG-3′; P. cynomolgi Rev, 5′-GGTATGATAAGCCAGGGA AGTG-3′; and probe, 5′ 6-carboxyfluorescein (FAM)-TACTCGCTCCTTCTGTTCCCTGGA-black hole quencher 1 (BHQ1)-3′. Real-time PCR was carried out in a total 25-μl reaction using a Rotor-Gene multiplex PCR kit (Qiagen) and a final concentration of primer and probe at 0.5 μm and 0.2 μm, respectively. PCR cycling conditions consist of a PCR initial activation step at 95°C for 5 min, followed by 45 cycles of denaturation at 95°C for 15 secs and annealing/extension at 60°C for 15 secs. The fluorescence data were acquired during the annealing/extension step. Blood from a macaque (R915) previously infected with P. cynomolgi was used as a positive control, and a cutoff at cycle 36 was used to define P. cynomolgi-positive samples in this study.

### Pharmacokinetics.

**(i) Sample collection and preparation.** Blood sampling (1 ml) for pharmacokinetic time points are as follows: just prior to the first ivermectin dose; after first dose at 1, 2, 4, 8, and 12 hours; each consecutive day just before dosing; and then after the 7th dose at 1, 2, 4, 8, and 12 hours and days 1, 2, 5, 12, and 19. If a primary infection occurred, then the same blood sampling schedule was repeated. No blood samples for pharmacokinetics were collected at first or second relapses. Blood was collected in heparinized sodium Vacutainer tubes and centrifuged at 2,500 rpm for 20 min, and then the supernatant (plasma) was transferred and kept at –80°C until analysis was performed. Plasma was separated into two tubes with 200 to 400 μl in each tube. Ivermectin was extracted using the protein precipitation method with 2:1 of acetonitrile (with internal standard):plasma volume, vortex mixed for 1 min, and then centrifuged at 10,000 rpm for 10 min. A total of 200 μl of supernatant fluid was filtered through a 0.22-μm polytetrafluoroethylene (PTFE) membrane prior to injection into a ultraperformance liquid chromatography (UPLC) system.

### (ii) Liquid chromatography-mass spectrometry analysis.

The liquid chromatography-mass spectrometry (LC-MS) was performed on an Acquity UPLC system equipped with a Xevo G2-XS quadrupole time of flight (QTOF) mass spectrometer (Waters Corp., Milford, MA, USA). A Waters Acquity UPLC ethylene-bridged hybrid (BEH) C_18_ column (50 by 2.1 mm, 1.7-μm particle size) with a precolumn of the same material was used to separate the compounds. The gradient mobile phase used for analysis of ivermectin was 5 mM ammonium formate and 0.1% formic acid in waters and methanol, with the column temperature of 40°C and flow rate at 0.4 ml/min. The total run time was 7 min, and the injection volume was 5 μl. Mass spectrometry was set in the positive electrospray ionization mode with multiple reaction monitoring. Instrument parameters included capillary voltage of 3.5 kV and source and desolvation temperatures of 150 and 400°C, respectively. The nitrogen generator was set at 120 lb/in^2^ to generate cone and desolvation gas flow rates of 50 and 800 liters/h, respectively. The mass transitions were observed at *m/z* of 892.77→569.50 and 894.79→571.52 for ivermectin and ivermectin-D2, respectively. Masslynx software (Waters Corp.) was used for quantification.

### (iii) Pharmacokinetic analysis.

Noncompartmental analysis (NCA) was used to generate pharmacokinetic parameters using Phoenix WinNonlin 8.1 (Certara USA, Inc., NJ, USA). The pharmacokinetic (PK) parameters determined were the elimination half-life (*t*_1/2_), maximum concentration of drug in serum (*C*_max_), time to *C*_max_ (*T*_max_), area under the concentration-time curve in 24 hours (AUC_24_), area under the concentration-time curve after the last dose to infinity (AUC_∞_), and percentage of AUC_∞_ due to extrapolation from last collection time point (*T*_last_) (AUC_%Extrap_), and since the fraction of dose absorbed cannot be estimated for extravascular models, apparent volume of distribution during terminal phase (*V_z_*/F) and apparent clearance (CL/F) were substituted for *V* and CL. Data analysis and graphical representation were completed using GraphPad Prism version 8.0.

### (iv) Pharmacokinetic modeling and simulation.

Generated NCA pharmacokinetic parameters were used as parameter estimates for compartment modeling. Observed ivermectin concentrations were best described by one-compartment analysis with first-order absorption and first-order elimination.

### Ethics statement.

The USAMD-AFRIMS Institutional Animal Care and Use Committee and the Animal Use Review Division, U.S. Army Medical Research and Materiel Command, reviewed and approved this study (PN 16-03). Animals were maintained in accordance with established principles under the Guide for the Care and Use of Laboratory Animals eighth edition (37) and the Animals for Scientific Purposes Act (38) and its subsequent regulations. The USAMD-AFRIMS animal care and use program is fully accredited by the Association for Assessment and Accreditation for Laboratory Animal Care International (AAALACi). Following the guide (37), animals enrolled in this study were part of the environmental enrichment program which aims to enhance animal well-being by providing the macaques with sensory and motor stimulation for facilitating the expression of species-typical behaviors and promoting psychological well-being. All macaques were housed under conditions that provide sufficient space in accordance with established rules and regulations. Macaques were housed individually; however, opportunity for direct and indirect contact with conspecifics was provided to maintain their social environment. Animal care and husbandry were provided throughout the study by trained personnel and under the direction of licensed veterinarians.
